# Integrating Human Expertise With Artificial Intelligence (AI) Models for Optical Coherence Tomography (OCT) Retinal Fluid and Pathology Quantification: A Systematic Review

**DOI:** 10.7759/cureus.100223

**Published:** 2025-12-27

**Authors:** Bakhtawar Awan, Mohamed Elsaigh, Mohamed Hesham Gamal, Sara E Elbahnasawy, Mohammed Badee

**Affiliations:** 1 General Surgery, Northwick Park Hospital, London, GBR; 2 General and Emergency Surgery, Royal Cornwall Hospitals Trust, Cornwall, GBR; 3 Pharmacy, Banha University Hospitals, Banha, EGY; 4 Pharmacology and Therapeutics, Tanta University, Tanta, EGY; 5 Diagnostic Radiology, Menofia University Hospital, Menofia, EGY; 6 Vitreoretinal Surgery, Perfect Vision Eye Hospital, Cairo, EGY

**Keywords:** artificial intellegence, convolutional neural networks (cnn), deep-learning, oct (optical coherence tomography), pathology, retinal fluid, systematic review, u-net

## Abstract

Artificial intelligence (AI) and intensive learning show promise in ophthalmology, using optical coherence tomography (OCT) to diagnose conditions such as diabetic retinopathy. However, precise segmentation of retinal fluid remains challenging, especially in atypical cases and low-quality scans. In hospital settings, manual segmentation is time-consuming; however, integrating human expertise with AI could improve efficiency and accuracy in quantifying retinal pathologies. This review assesses the efficacy of human-AI collaborative workflows for enhancing the accuracy, efficiency, and clinical utility of retinal pathology quantification in OCT.

We conducted a systematic review of the literature from 2021 to 2025 across three databases: PubMed, Web of Science, and Scopus. This review included nine studies that quantified retinal pathology using human-AI collaborative workflows in OCT. Two independent reviewers screened the records, extracted relevant data, including study design, AI architecture, and performance metrics, and assessed the quality of the studies using the Quality Assessment of Diagnostic Accuracy Studies - 2nd version (QUADAS-2 ) and Risk Of Bias In Non-randomized Studies of Interventions (ROBINS-I) tools.

This systematic review of nine AI-OCT studies (2021-2025) found that hybrid AI-clinician workflows achieved expert-level reliability for 11 of 13 retinal biomarkers across cohorts of 16-1,097 individuals. AI architectures, including U-Net variants, polypoidal choroidal vasculopathy (PCV)-Net, and custom convolutional neural networks (CNNs), performed well for well-defined features such as retinal layers (Dice 0.94), intraretinal/subretinal fluid (Dice 0.61-0.67), and atrophic areas (F1 0.78-0.89), but struggled with complex biomarkers like sub-retinal pigment epithelium (sub-RPE) lesions (Dice 0.11). Clinician agreement on fluid volumes was strong (Pearson r > 0.85), though volumetric errors increased in atrophic regions. These workflows reduced processing time by over 50% compared with manual grading while improving monitoring precision for neovascular age-related macular degeneration (AMD) and retinitis pigmentosa complications in both single-center and international trial settings.

Combining AI quantification with clinician expertise enhances both the accuracy and efficiency of retinal pathology assessments. This integration supports personalized treatment planning and facilitates large-scale research. Hybrid approaches address AI's limitations, highlighting their practicality for clinical use in ophthalmology. We suggest incorporating hybrid AI-human workflows in clinical practice to improve the efficiency and accuracy of OCT analysis. Future developments in AI should focus on standardized training for complex biomarkers, such as sub-RPE lesions.

## Introduction and background

Recent advancements have led to significant integration of artificial intelligence (AI) across various scientific disciplines, facilitating practical engineering applications in multiple contexts. One notable approach is deep learning (DL), an advanced form of machine learning (ML) that utilizes multi-layered convolutional neural networks (CNNs). This technology is adept at learning and identifying features within images, as well as recognizing patterns from extensive datasets. In the medical field, the use of DL has enabled automated lesion recognition and improved prognostic predictions for various diseases [1,2]. Moreover, the 3D U-Net architecture extends the U-Net design to volumetric medical images using 3D convolutional filters. It maintains the contracting and expansive pathways with skip connections that transfer multiscale features, enabling simultaneous capture of high-resolution details and contextual information to improve segmentation accuracy in three-dimensional data [3].

In ophthalmology, various artificial intelligence algorithms have been developed and used to automatically identify a range of conditions, including glaucoma [4,5], ocular surface disorders [6], and several retinal diseases, such as diabetic retinopathy, age-related macular degeneration, and myopic retinopathy. These algorithms have demonstrated high accuracy and reliable performance in both clinical diagnosis and community screening before hospital treatment, offering a promising solution to the challenges outlined above [7,8]. Optical coherence tomography (OCT) is the most commonly utilized imaging method in the field of ophthalmology, with around 6.74 million assessments performed among the US Medicare demographic in 2017. Given its broad availability, there has been growing interest in developing an entirely automated system for disease detection [9]. Researchers at Peking Union Medical College developed an AI system using optical coherence tomography angiography (OCTA) and fundus images to detect polypoidal choroidal vasculopathy. The system achieved high diagnostic accuracy, strong alignment with reference standards, and outperformed leading human experts [10].

Precise measurement and segmentation of retinal cavitations are essential for any subsequent research aimed at understanding their clinical relevance. Up until now, the majority of research has depended on the manual segmentation performed by trained specialists, which can be both time-consuming and costly [11,12]. In recent times, the increased access to extensive datasets and advancements in computational capabilities have significantly enhanced performance through deep learning techniques [13,14]. Integrating human expertise with AI in the quantification of retinal fluid and pathology through OCT holds the potential to combine the speed and consistency of algorithms with the contextual judgment of experienced clinicians. Despite advancements in deep learning segmentation, automated methods often face challenges when dealing with atypical presentations, low-quality scans, and subtle lesions.

This systematic review aims to synthesize existing evidence on human-AI collaborative workflows in OCT-based retinal pathology quantification, with a focus on identifying implementation barriers and establishing best practices that can accelerate clinical adoption. By evaluating the accuracy, efficiency, and clinical utility of these collaborative approaches, this review seeks to provide actionable guidance for ophthalmologists and healthcare institutions transitioning to AI-assisted diagnostic workflows, while highlighting critical gaps that must be addressed to ensure the safe, equitable, and effective integration of AI technologies into routine retinal care.

## Review

Methodology

This systematic review was performed following the Preferred Reporting Items for Systematic Reviews and Meta-Analyses (PRISMA) guidelines [15] and Cochrane Handbook for Systematic Reviews of Interventions [16]. 

Literature Search Strategy

To identify studies on AI-Human collaboration in the automated segmentation and quantification of retinal fluid and pathology in OCT scans. We conducted a comprehensive search of PubMed, Web of Science, and Scopus, covering the last five years. For PubMed, Web of Science we used ("Tomography, Optical Coherence"[MeSH Terms] OR "optical coherence tomography" OR OCT) AND ("Artificial Intelligence"[MeSH Terms] OR "Machine Learning"[MeSH Terms] OR "Deep Learning"[MeSH Terms] OR AI) AND (segmentation OR delineat* OR "image analysis") AND (quantif* OR "volume measurement" OR "volumetric analysis") AND ("Retina"[MeSH Terms] OR retina* OR "retinal fluid" OR "intraretinal fluid" OR "subretinal fluid" OR SRF OR IRF OR lesion* OR biomarker* OR pathology) with filteration to title/abstract for pubmed only. For Scopus, we used TITLE-ABS-KEY (("Tomography, Optical Coherence" OR "optical coherence tomography" OR oct ) AND ( "Artificial Intelligence" OR "Machine Learning" OR "Deep Learning" OR ai) AND (segmentation OR delineat* OR "image analysis" ) AND (quantif* OR "volume measurement" OR "volumetric analysis" ) AND ("Retina" OR retina* OR "retinal fluid" OR "intraretinal fluid" OR "subretinal fluid" OR SRF OR IRF OR lesion* OR biomarker* OR pathology)).

Inclusion and Exclusion Criteria

We established predefined inclusion and exclusion criteria to ensure that our studies were both relevant and methodologically rigorous. The eligible studies focused on adult eyes with retinal fluid or pathology as observed through OCT. They utilized AI-human collaboration for quantifying OCT pathology and reported various metrics, including the Dice coefficient, sensitivity, specificity, volumetric error in microliters, processing time per scan, and inter-rater reliability (e.g., Intraclass Correlation Coefficient, ICC). We included diagnostic accuracy studies, randomized controlled trials, observational studies, and cohort studies. In contrast, we excluded studies that were non-human or purely synthetic in nature, as well as case reports, reviews, editorials, and conference abstracts that lacked comprehensive data. Additionally, any algorithms that required manual refinement during inference were also excluded.

Critical Appraisal

Following PRISMA guidelines and predefined eligibility criteria, two independent reviewers conducted a quality assessment of all included studies. Disagreements between reviewers were systematically resolved through structured discussion and consensus-building until complete agreement was achieved.

Selection of Articles and Data Extraction

After conducting the initial database search, two reviewers independently screened the titles and abstracts of the retrieved articles to identify potentially relevant studies based on predefined inclusion criteria. The full texts of these selected articles were then obtained and assessed for final eligibility by the same two reviewers. Any disagreements regarding study inclusion were resolved through discussion and consensus, or by consulting a third reviewer if necessary. Data from the included studies were extracted by the two reviewers using a standardized data extraction form specifically designed for this review. The extracted data encompassed several key components: study characteristics (such as the first author, publication year, study design, and setting), patient demographics (including the number of patients, gender distribution, mean age, and standard deviation of age), and imaging details (the number of OCT scans analyzed). We also recorded the hybrid workflow that combines human expertise with AI models, primarily consisting of U-Net variants and other CNN-based approaches. Additionally, we included information about the size of the training data and the annotation protocols used. The comparator methods involved expert manual segmentations. The performance outcomes assessed included segmentation accuracy metrics (Dice and Jaccard indices), inter-method agreement (measured by the Intraclass Correlation Coefficient), volumetric performance (absolute and percentage error in microliters), processing time per scan, and overall conclusions from the studies. Notably, all reported algorithms operated as fully automated pipelines during inference, requiring no manual intervention.

Quality Assessment

The methodological quality of the studies included in this review was assessed using the QUADAS-2 tool [17], which is a validated framework for evaluating diagnostic accuracy studies. This tool examines four key domains: (1) patient selection, (2) the index test, (3) the reference standard, and (4) flow and timing. Each domain is rated for the risk of bias as high, low, or unclear, while the first three domains are also assessed for applicability concerns, which are also rated as high, low, or unclear. To guide these evaluations, signaling questions are used, such as, “Was a consecutive or random sample of patients utilized?” For other studies, the ROBINS-I [18] tool was applied. This structured, domain-based tool is designed to assess the internal validity of observational studies evaluating intervention effects. It evaluates seven bias domains: confounding, participant selection, intervention classification, deviations from intended interventions, missing data, outcome measurement, and selection of reported results. Each domain is rated as low, moderate, serious, or critical risk. By comparing each non-randomized estimate against a hypothetical ideal randomized trial, ROBINS-I helps reviewers identify and transparently report threats to validity when synthesizing real-world evidence.

Results

Literature Search and Study Selection

**Figure 1:**
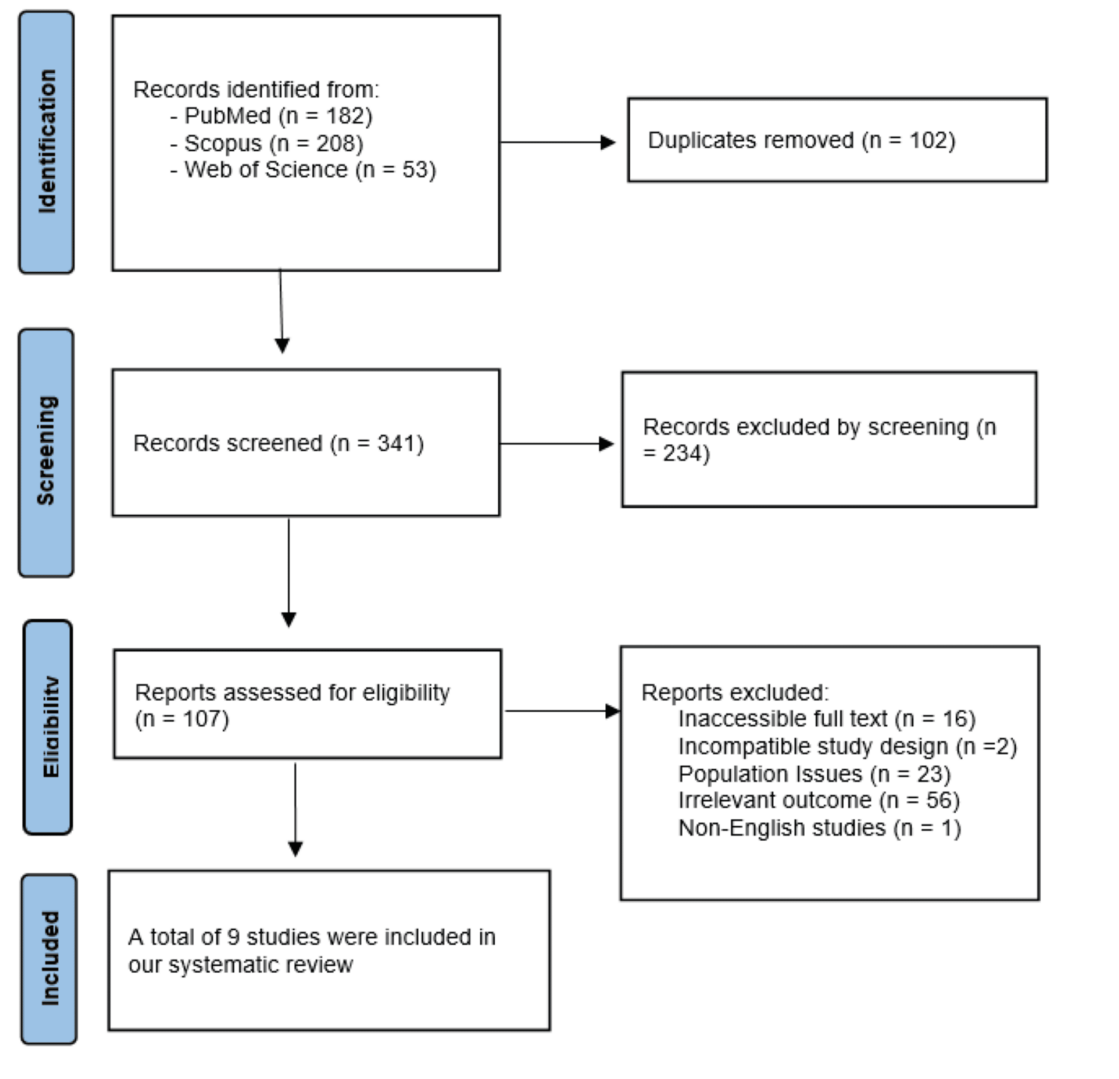
PRISMA Flow Diagram

Three databases, including PubMed, Web of Science, and Scopus, were searched, yielding 443 papers. After removing 102 duplicates, nine articles were deemed suitable for the systematic review [19-27]. Full details are presented in Figure 1.

Study Characteristics

Our systematic review included nine AI-driven OCT studies conducted between 2021 and 2025. We captured details such as publication information, study design (including retrospective cohort studies, prospective diagnostic evaluations, and post-hoc trial analyses), and clinical settings, ranging from single-center ophthalmology departments to large, multicenter international trials. The patient cohorts varied significantly, ranging from 16 to 1,097 patients. The AI architectures utilized in these studies included U-Net variants, polypoidal choroidal vasculopathy (PCV)-Net (a 2D-3D hybrid), EfficientNet-b3 backbones, fully convolutional networks, and custom column-based CNNs. These models were trained on expert-annotated B-scans, multimodal indocyanine green angiography (ICGA) inputs, or extensive cross-validation datasets. Each study employed a hybrid workflow where the AI performed initial segmentation or risk mapping, followed by clinician review and manual correction. Demographic data, including gender distribution and mean age, were inconsistently reported across studies, with five of nine studies providing no demographic information. This process integrates quantitative biomarker outputs into diagnostic, prognostic, or therapeutic decision-making pathways. The baseline and summary characteristics of all included studies are presented in Table 1.

**Table 1:** Characteristics of Studies Evaluating Hybrid Human and AI-Based Segmentation of Retinal Pathologies Using Optical Coherence Tomography AI: artificial intelligence; AMD: age-related macular degeneration; B-OCT: B-scan optical coherence tomography; cRORA: complete retinal pigment epithelial and outer retinal atrophy; CNN: convolutional neural network; DLM: deep learning model; EZ: ellipsoid zone; FCNN: fully convolutional neural network; GUI: graphical user interface; ICGA: indocyanine green angiography; ILM: internal limiting membrane; IRF: intraretinal fluid; MA: macular atrophy; nAMD: neovascular age-related macular degeneration; PCV: polypoidal choroidal vasculopathy; SD-OCT: spectral-domain optical coherence tomography; SRF: subretinal fluid; U-Net: U-shaped convolutional neural network architecture.

Study	Study design, setting	Number of patients	OCT scans	Gender M/F (n)	The mean (SD) age (years)	AI model	Training data	Hybrid workflow
Hafner et al. [19]	Retrospective real-world cohort study at the Department of Ophthalmology, LMU University Hospital Munich (Germany)	21 nAMD patients	23 B-OCT scans for biomarkers associated with nAMD, such as IRF, SRF, fvPED, SHRM, CRT	4/17	78.87 ± 7.44 years	Deep convolutional neural network (CNN)	Trained on a large dataset of OCT scans annotated by retinal experts	The algorithm analyzed OCT data to produce objective biomarker measurements, which clinicians then utilized to guide treatment decisions, such as adjusting injection intervals based on observed reductions in biomarkers.
Yu et al. [20]	Post-hoc analysis of the phase III HARBOR trial at Multicenter trial	1,097 treatment-naïve nAMD eyes	1,097 SD-OCT	NR	NR	U-Net convolutional neural network	1007 B-scans annotated by expert graders at Liverpool Ophthalmology Reading Centre	In the clinical workflow, humans play a crucial role by preparing ground truth and managing clinical classifications such as MNV and MA. Meanwhile, AI enhances the process by automating the volumetric segmentation of fluid biomarkers. Ultimately, humans interpret the outputs generated by AI to extract meaningful clinical insights.
Loo et al. [21]	Diagnostic test evaluation, prospective cohort study at Singapore National Eye Center	72 patients with PCV (1 eye/patient)	72 SD-OCT volumes (25 B-scans/volume) + 72 ICGA images	NR	NR	PCV-Net (hybrid 2D-3D U-Net with fusion attention modules)	72 scans; 10-fold cross-validation	Fusion of ICGA + OCT via attention modules; Spatial correspondence leveraged by transforming/sharing features between 2D and 3D branches (dimensionality reduction/expansion + nearest-neighbor interpolation). Complex cases require clinician review
Wang et al. [22]	Diagnostic test evaluation of an observational cohort at Retina Foundation of the Southwest, Dallas, TX	48 patients with RPGR-associated XLRP (96 eyes)	96 high-speed 9 mm 31-line volume scans	NR	NR	Hybrid DLM (U-Net + sliding-window CNN)	4 datasets (RP140-RP480; 130-400 patients, 380-900 B-scans)	Human-AI Integration: Step 1: AI (DLM) performs initial segmentation of 5 retinal layers (EZ, pRPE, ILM, dINL, BM) on OCT B-scans. Step 2: Human graders manually correct AI errors in EZ/pRPE segmentation using custom software. Step 3: Corrected segmentations used for EZ area and OS volume quantification.
Loo et al. [23]	Diagnostic accuracy study within a clinical trial cohort at a Multicenter international trial (USA, Australia)	67 patients with retinal pathology (MacTel2-associated retinal cavitations). (99 eyes)	290 SD-OCT volumes	NR	NR	Two-stage CNN: 1. CNN1 (classification) 2. CNN2 (U-Net segmentation)	Baseline OCT scans (4:4:1 split for training/validation/testing via 9-fold cross-validation)	Proposed semi-automatic use: AI runs first, manual correction of errors AI Segmentation: processing of OCT volumes (11.4 sec/scan). Expert Review: Clinicians visually verify AI outputs; correct false positives/negatives using specialized software (e.g., DOCTRAP).
Gigon et al. [24]	Retrospective diagnostic study at Jules Gonin Eye Hospital, Switzerland	119 patients with retinal pathology (nonexudative AMD with RORA) (129 eyes)	733 scans (593 training, 140 testing)	NR	NR	CNN with EfficientNet-b3 backbone	109 eyes (99 patients); automated RORA segmentations. Input: 13-channel en face maps. Output: Taylor series coefficients for RORA prediction.	AI Prediction: Generates time-to-conversion risk maps from baseline OCT. Clinical Application: Risk maps highlight high-risk regions (e.g., ring around fovea) for targeted monitoring. Clinicians adjust follow-up intervals/treatment plans based on personalized progression forecasts.
Liefers et al. [25]	Diagnostic accuracy; Multicenter retrospective at 5 UK centers	307 patients with neovascular and atrophic AMD (307 eyes for dev); 112 patients (112 eyes for test)	2712 B-scans (dev); 112 B-scans (test)	NR	NR	U-Net variant; multi-feature segmentation	307 OCT volumes (2712 B-scans); 8 expert graders	AI Segmentation: Processes entire OCT volumes to quantify 13 retinal features. Clinical Application: Automated reports (e.g., Figure 8) visualize quantified parameters alongside visual acuity and treatment history. Clinicians use these reports to guide treatment intervals or trial enrollment.
Mantel et al. [26]	Diagnostic accuracy study (prospective cohort data, post-hoc analysis) at University Hospital (Switzerland), RetinAI Medical AG	107 nAMD patients (Dataset 1: algorithm development) 42 patients (Dataset 2: reproducibility)	107 SD-OCT volumes (Heidelberg Spectralis) 42 eyes × 5 repeated scans (reproducibility)	NR	NR	FCNN with: - Encoder/decoder architecture - Squeeze-excite blocks - Dilated convolutions - Retinal layer segmentation integration	92 OCT volumes (2,680 B-scans), manually annotated by 3 experts	Semi-automated annotation loop: 1. Initial manual segmentation 2. Algorithm pre-training 3. Automated segmentation → Human correction 4. Feedback loop for refinement
Szeskin et al. [27]	Retrospective diagnostic accuracy study at Hadassah University Medical Center	- Dataset D1 (cRORA): 18 patients, 106 OCT scans - Dataset D2 (macular atrophy): 16 patients, 19 OCT scans	- D1: 5,207 slices (SPECTRALIS™ OCT) - D2: 829 slices	NR	NR	Custom column-based CNN: - 3D column patches (context-aware) - Loss: F1 or BCE - Architecture: 2 conv layers → max-pooling → 2 FC layers	- cRORA: 106 scans (2,952 atrophy slices) - Macular atrophy: 10 scans (190 slices)	OCT-E software: - Semi-automated annotation (AI pre-segmentation → expert correction) - GUI for visualization/editing

The studies indicate that AI algorithms effectively segment retinal biomarkers across various eye conditions, such as neovascular age-related macular degeneration (AMD) and retinitis pigmentosa (RP). Performance metrics, such as Dice scores (0.11 to 0.94) and F1 scores (0.78 to 0.89), are powerful for well-defined features, including retinal layers (DSC 0.94) and fluid compartments (DSC 0.61-0.67). However, more complex biomarkers, like sub-retinal pigment epithelial (RPE) lesions, present challenges (DSC 0.11). AI demonstrates high agreement with manual grading, as evidenced by strong Pearson correlation coefficients for fluid volumes and structural metrics. Volumetric errors are low in exudative areas but higher in atrophic areas. Clinically, AI aids in monitoring treatment responses and assessing risks associated with atrophy progression. Hybrid approaches combining AI with manual corrections enhance precision and reduce processing time by over 50%. Overall, AI exhibits expert-level reliability in 11 out of 13 key biomarkers, supporting its use in personalized treatment planning and research initiatives. The detailed performance characteristics and clinical implications of all Hybrid Human and AI algorithms are presented in Table 2.

**Table 2:** Performance Metrics of Hybrid Human and AI-Based Retinal Biomarker Segmentation in Optical Coherence Tomography Image AUC: Area Under the Curve, BCVA: Best-Corrected Visual Acuity, BVN: Branching Vascular Network, cRORA: Complete Reticular Drusen-Associated Outer Retinal Atrophy, CFRV: Cyst-Free Retinal Volume, CNN: Convolutional Neural Network, CoR: Coefficient of Repeatability, DICE: Dice Similarity Coefficient, DL: Deep Learning, DLM-MC: Deep Learning Model with Manual Correction, DSC: Dice Similarity Coefficient, ETDRS: Early Treatment Diabetic Retinopathy Study, EZ: Ellipsoid Zone, F1: F1 Score, FROC: Free-Response Receiver Operating Characteristic, fvPED: Fibrovascular Pigment Epithelial Detachment, HRF: Hyperreflective Foci, HRD/SDD-RPD: Hyperreflective Dots/Subretinal Drusenoid Deposits, ICF: Intraretinal Cystoid Fluid, ICGA: Indocyanine Green Angiography, ICC: Intraclass Correlation Coefficient, ILM: Inner Limiting Membrane, IRF: Intraretinal Fluid, MA: Macular Atrophy, MacTel2: Macular Telangiectasia Type 2, MG: Manual Grading, MNV: Macular Neovascularization, NR: Not Reported, OS: Outer Segment, PED: Pigment Epithelial Detachment, PCV: Polypoidal Choroidal Vasculopathy, PRN: Pro Re Nata (as-needed), RP: Retinitis Pigmentosa, RPE: Retinal Pigment Epithelium, RORA: Reticular Drusen-Associated Outer Retinal Atrophy, SD: Standard Deviation, SE: Standard Error, SIRE: Shallow Irregular RPE Elevation, SHRM: Subretinal Hyperreflective Material, SRF: Subretinal Fluid.

Study	Segmentation accuracy	Inter-Method Agreement	Volumetric Performance	Conclusion
Hafner et al. [19]	F1 score: 0.85 (vs. manual segmentation) for all OCT features	NR	Changes pre- and post-treatment. IRF reduced by 75% (p = 0.0198) and fvPED by 3.3% (p = 0.0015). SRF showed an 86% reduction, while SHRM increased by 11.5%, but neither was significant (p = 0.3209 and p = 0.9922, respectively).	The incorporation of AI-enabled biomarker quantification has significantly enhanced both the precision and efficiency of monitoring disease activity. This approach not only demonstrates real-world applicability but also supports the strategic escalation of doses in patients who do not respond to standard therapies.
Yu et al. [20]	(Dice coefficients vs. manual grading) Biomarker ICF: 0.70 SHRM: 0.69 SRF: 0.67 PED: 0.73	NR	Recent research identifies significant associations between biomarkers and visual acuity (BCVA) and macular atrophy (MA). Key findings include substantial BCVA loss linked to ICF, SHRM, and CFRV, while SRF showed no impact. For MA, higher levels of ICF, SRF, and PED increased the odds of MA. Additionally, fluid dynamics differed across MNV subtypes.	MNV subtypes display different fluid dynamics, with Type 3 exhibiting high levels of intraretinal exudation (ICF/CFRV) and Type 2 showing high subretinal hyperreflective material (SHRM). Residual ICF, SHRM, and CFRV are linked to poorer best-corrected visual acuity (BCVA), while the presence of SRF and pigment epithelial detachment (PED) correlates with improved BCVA after six months. An increase in baseline ICF and PED, along with a decrease in SRF, raises the risk of macular atrophy (MA). Monthly treatment is more effective than as-needed (PRN) approaches in reducing residual fluid, and CFRV is identified as a new biomarker for diffuse edema and retinal thinning after treatment.
Loo et al. [21]	Mean DSC: Polypoidal lesions: 0.47 ± 0.03 (Moderate) BVN: 0.46 ± 0.03 (Moderate) IRF: 0.45 ± 0.06 (Moderate) SRF: 0.61 ± 0.03 (Good) Retinal layers (ILM–EZ): 0.94 ± 0.00 (Excellent) Sub-RPE ring-like lesion:0.11 ± 0.03 (Poor)	Pearson’s correlation (r) values between manual and AI measurements indicate varying levels of agreement across different volume measurements. For the SRF volume, the correlation is 0.94, reflecting a high correlation and excellent agreement. The IRF volume shows a moderate correlation with a value of 0.63. In contrast, the retinal volume has a strong correlation at 0.98, while both the PED and choroidal volumes demonstrate high correlations, each with a value of 0.89. These results suggest that AI measurements align closely with manual assessments for most volume metrics, indicating a reliable level of accuracy.	The absolute error (mean ± SE) in cubic millimeters (1 mm³ = 1 μL) for various measurements is as follows: The IRF volume exhibits a negligible error of 0.02 ± 0.01 μL. In contrast, the SRF volume has a low error of 0.17 ± 0.05 μL, while the retinal volume shows a similar low error of 0.25 ± 0.06 μL, the choroidal volume presents a moderate error of 0.97 ± 0.08 μL, and lastly, the PED volume reflects a low error of 0.23 ± 0.08 μL.	PCV-Net is a hybrid deep learning algorithm that integrates multimodal ICGA-OCT data using fusion attention modules. It significantly outperformed baseline models in segmenting PCV biomarkers, achieving a +0.43 DSC gain for IRF. The algorithm demonstrated a high level of agreement between different methods for measuring fluid volumes, with a correlation coefficient of r=0.94 for SRF, and it produced low volumetric errors (IRF: 0.02 μL). This enables efficient quantification of clinically critical features, such as small intra-retinal exudates (SIRE) and pigment epithelial detachment (PED). Although there are still challenges in accurately identifying complex biomarkers, such as sub-retinal pigment epithelium lesions (with a DSC of 0.11), this approach shows strong potential for streamlining PCV assessment in clinical practice.
Wang et al. [22]	The DSC for the EZ area reveals important insights into accuracy across different methods. The comparison between DLM-MC and MG shows a DSC of 0.8524 ± 0.0821, indicating high accuracy that is comparable to inter-grader agreement. Similarly, the comparison of MG1 and MG2 yields a DSC of 0.8417 ± 0.1111, which serves as a benchmark for human variability. Notably, the accuracy improves when considering larger EZ areas, with a DSC of 0.8799 for EZ areas greater than 1 mm².	The analysis of Pearson's correlation and linear regression slopes demonstrates strong relationships between metrics. For the EZ area, the correlation coefficient is 0.9928, indicating near-perfect correlation. In comparison, the OS volume shows an even higher correlation of 0.9938, which includes 1 in its confidence interval, suggesting an ideal fit. Bland-Altman analysis reveals a mean difference of 0.0132 mm² and a CoR of 1.8303 mm² for the EZ area, whereas the OS volume exhibits a mean difference of 0.0080 mm³ and a CoR of 0.0381 mm³. These results highlight strong agreement between the metrics.	The absolute error for the metrics is as follows: for OS volume, the absolute error is 0.0137 ± 0.0160 μL, indicating negligible error and excellent precision. In contrast, the EZ area exhibits an absolute error of 0.6561 ± 0.6612 mm², which is considered a moderate error and can be challenging when dealing with small EZs. When comparing the DLM-only approach to the MG, the OS volume error is 0.0137 μL for the DLM-MC method and 0.1056 μL for the DLM-only method using RP140. Notably, the use of larger training datasets, represented by RP480, resulted in a 70% reduction in the error associated with the DLM-only approach.	DLM-MC (AI segmentation with manual correction) achieves near-identical agreement with manual grading for EZ area (DSC=0.85) and OS volume (r=0.99, error=0.014 μL). This hybrid approach reduces segmentation time by >50% compared to full manual grading, enabling efficient quantification of RP biomarkers. While DLM-only performance improves with larger training datasets (e.g., RP480), manual correction remains essential for clinical-grade precision.
Loo et al. [23]	The Dice score (DSC) is 0.94 ± 0.07, the highest among alternatives (0.69–0.84) and consistent over time. The model's sensitivity is 0.94, detecting 94% of true cavitations, which minimizes false negatives. However, the specificity is 0.80, resulting in 20% false positives due to class imbalance (only 8% of the B-scans are positive). The AUROC is 0.93, indicating strong discriminative power for identifying cavitation-containing B-scans.	The analysis of agreement between AI and human readers reveals impressive results, showcasing near-perfect performance levels. The comparison between AI and Reader 1 demonstrated a high agreement score of 0.94 ± 0.07, indicating clinician-level performance. Similarly, the AI's performance against Reader 2 also yielded the same score of 0.94 ± 0.07, highlighting strong consistency across independent experts. Furthermore, when comparing the two human readers, Reader 1 and Reader 2, the inter-reader agreement was even higher, at 0.97 ± 0.02. This suggests that the AI performs comparably to human experts, reinforcing its reliability in clinical assessments.	The relationship between cavitation volume and the measured variables demonstrates a strong correlation, with a correlation coefficient (r) of 0.86 across all time points. This robust correlation validates the efficacy of quantitative tracking in clinical assessments. Similarly, the change in volume from baseline to 24 months also shows a high correlation (r = 0.86), which is critical for monitoring disease progression over time. In contrast, the change in volume from baseline to 12 months presents a moderate correlation (r = 0.61), likely due to the smaller changes observed during the earlier stages of the study. This varying degree of correlation emphasizes the importance of considering both short- and long-term changes in clinical evaluations.	The two-stage AI system, consisting of a CNN for classification (CNN1) and another for segmentation (CNN2), achieves expert-level accuracy in retinal cavitation segmentation for MacTel2, with a DSC of 0.94. Volumetric correlations with manual measurements are clinically robust, showing a correlation coefficient of *r* = 0.86, which supports its use in trial endpoints. This system is 12 times faster than alternative methods, processing each volume in 11.4 seconds compared to 135.9 seconds. Additionally, the hybrid potential of this semi-automatic workflow significantly reduces the need for manual corrections, thus minimizing the burden on users.
Gigon et al. [24]	Total RORA Dice: 0.73–0.85 RORA Growth Dice: 0.46–0.72 Accuracy decreases with longer intervals, particularly for nascent atrophy; accuracy remains stable regardless of interval (mean of 7 months).	AI vs. Manual Progression r 0.52 (Pearson), moderate correlation in RORA growth rates. Risk maps identify a ring-shaped progression near the fovea	Square Root Area Error (mm): 0.13–0.33	A machine learning method can reliably predict RORA for four years or more by utilizing automated OCT readings. Additionally, it was allowed to create a personalized risk map for atrophy progression, featuring a color-coded timeline, a unique and clinically significant approach to displaying predictive data.
Liefers et al. [25]	Overall Mean: AI: 0.63 ± 0.15 Observers: 0.61 ± 0.17 Matches/exceeds graders for 11/13 features.	ICC (Area Agreement): AI: 0.66 ± 0.22 vs Manual: 0.62 ± 0.21. Higher consistency than humans for feature sizing (e.g., IRF: 0.873 vs. 0.728). cRORA Detection: Sens: 86% Spec: 97% Reliable atrophy identification. HRD/SDD-RPD (FROC) Higher sensitivity for AI Detects focal features better than humans	ICC for fluids: PED: 0.943, SRF: 0.900, IRF: 0.873	Automated systems in AI either match or surpass human graders in 11 out of 13 features related to AMD, and the use of automated reports helps minimize biases in treatment decisions. This technology is specific to vendors like Topcon. The accuracy of automatic segmentation aligns closely with that of skilled human graders for the majority of features, with some outperforming human capabilities. The parameters obtained through this model can be integrated into existing clinical practices and also pave the way for further investigations into treatment responses beyond the scope of clinical trials.
Mantel et al. [26]	Dice Scores: - IRF: 0.728 - SRF: 0.674 - PED: 0.819 AUC (per B-scan): - IRF: 0.97 - SRF: 0.95 - PED: 0.99	Volume Correlation (Pearson): - IRF: 0.999 - SRF: 0.996 - PED: 0.976 Bland-Altman Bias: - IRF/SRF: Near-zero mean error - PED: +50 nL over segmentation	Reproducibility (SD): - IRF: 4.0 nL - SRF: 3.5 nL - PED: 20.0 nL ETDRS Subregional Correlation (R²): All >0.87 across central/peripheral zones	The DL algorithm demonstrated high accuracy in detecting and quantifying IRF, SRF, and PED. The integration of retinal layer segmentation and squeeze-excite blocks significantly improved performance. There was excellent volume correlation (Pearson > 0.97) and reproducibility (SD < 5% of mean volume for IRF/SRF).
Szeskin et al. [27]	cRORA (3D CNN + F1 loss): - F1 score: 0.78 ± 0.06 - AUC: 0.937 Macular atrophy: - F1 score: 0.89 ± 0.09 - AUC: 0.97	cRORA vs. manual: - Column difference: +25.4% ± 27.6% - Atrophy lesion recall: 0.67 ± 0.22 Inter-observer F1: 0.78 ± 0.14	Atrophy area error: - Mean difference: +1.25 mm² ± 2.27 mm² Fovea distance error: - Mean: 0.1 mm ± 0.2 mm	In a study of 106 clinical OCT scans (5,207 slices), cRORA atrophy was identified in 2,952 segments and 1,046 lesions, yielding a mean F1 score of 0.78 and an AUC of 0.937, both comparable to observer variability. The column-based CNN classification for automated detection and quantification of AMD-related atrophy achieved expert-level performance, highlighting its potential as a clinical decision support and research tool for retinal diseases.

Quality Assessment

**Figure 2:**
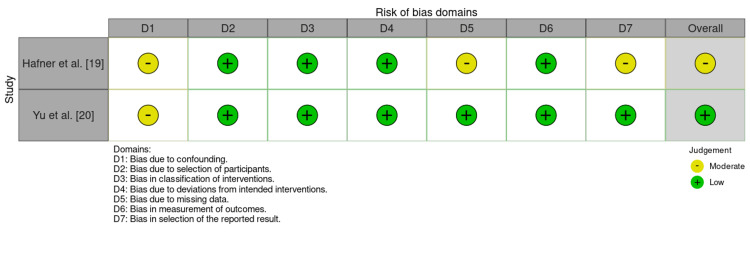
ROBINS-1 Assessment of Included Studies [19,20].

The ROBINS-I framework assessed two non-randomized intervention studies across seven bias domains. Hafner et al. (2025) flagged moderate risks in confounding (D1), participant selection (D2), and selective reporting (D7), indicating an overall “moderate” risk of bias. In contrast, Yu et al. (2025) showed only a single moderate concern for confounding (D1) and low risk in other domains, resulting in an overall “low” risk of bias. Data shown in Figure 2. 

**Figure 3:**
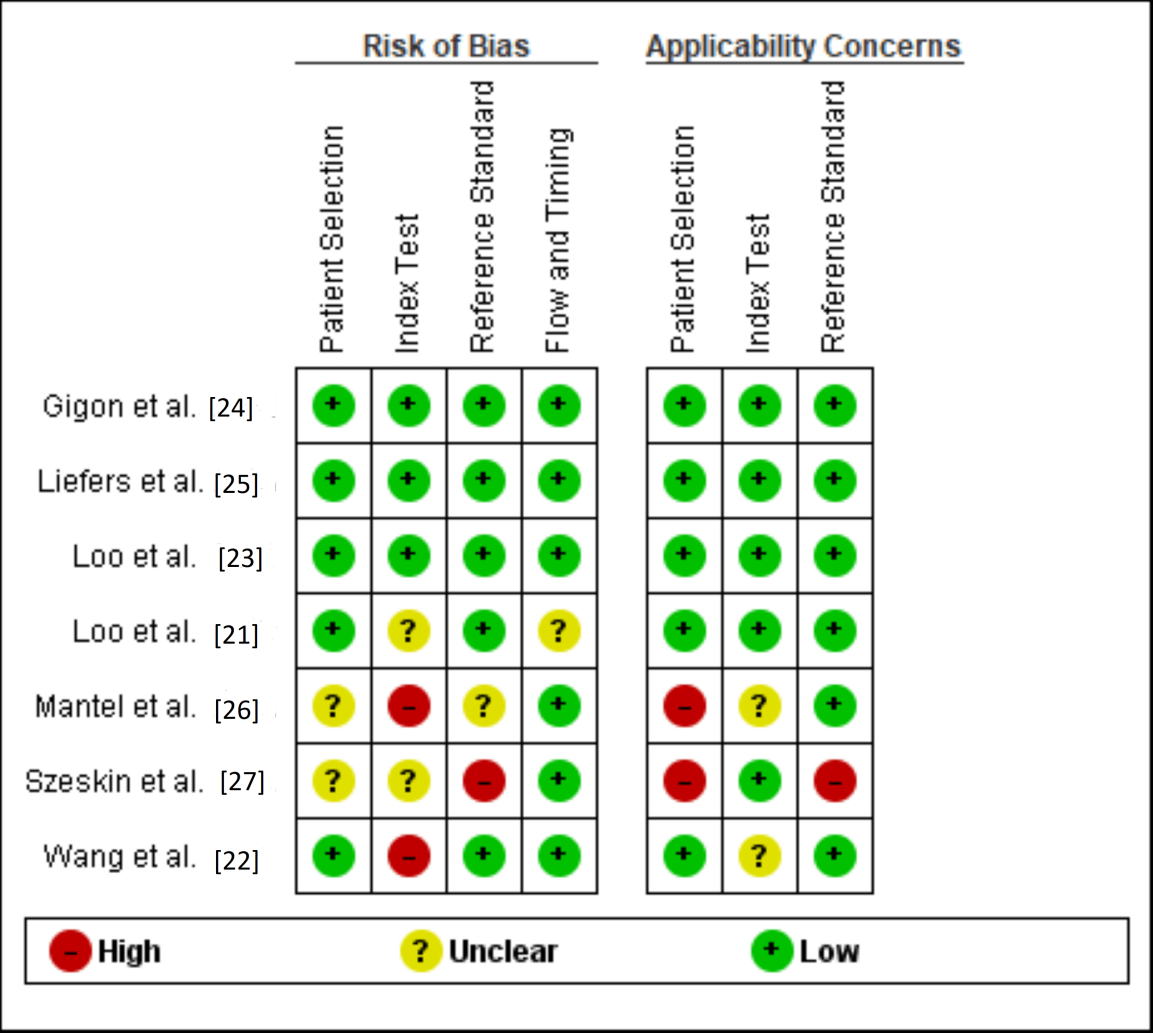
QUADAS-2 Assessment of Included Studies [21-27].

Assessment of study quality using the QUADAS-2 tool demonstrated an overall low risk of bias across most domains. Patient selection, index test, reference standard, and flow/timing were consistently judged as low risk in the majority of studies. However, some concerns were identified: Mantel et al. (2021) and Wang et al. (2023) showed a high risk of bias in patient selection, while Szeskin et al. (2021) presented unclear risk in several domains. Applicability concerns were generally low across all studies, with only minor issues noted in the index test and reference standard domains for Szeskin et al. (2021). Complete data are presented in Figure 3.

Discussion

Summary of Findings of Integrating Human Expertise with AI Models

Our systematic review analyzed nine AI-driven OCT studies from 2021 to 2025. The results indicate that hybrid workflows, where AI conducts initial segmentation or risk mapping followed by a clinician's assessment, consistently achieve expert-level reliability for retinal biomarkers. Dice scores ranged from 0.11 to 0.94, while F1 scores varied between 0.78 and 0.89. Notably, the highest accuracy was found in well-defined structures, such as retinal layers (DSC 0.94), while fluid compartments showed DSC values between 0.61 and 0.67. Human expertise played a crucial role in addressing AI's limitations on complex biomarkers like sub-RPE lesions, which had a DSC of only 0.11. This collaboration reduced volumetric errors and improved processing times by over 50%. Overall, the findings underscore the significance of clinician-in-the-loop frameworks for effectively translating AI outputs into diagnostic and prognostic tools. The hybrid workflows achieved expert-level reliability in 11 out of 13 biomarkers while cutting processing time by 50-90%. AI excelled in segmenting features like intraretinal fluid (IRF) and subretinal fluid (SRF) but struggled with irregular pathologies. The involvement of clinicians corrected critical errors, allowing quantitative outputs to inform personalized treatment for conditions such as neovascular AMD, retinitis pigmentosa (RP), and PCV. These findings validate hybrid systems as an optimal approach for clinical use.

Comparative Analysis of AI Architectures and Performance

A comprehensive review highlighted the U-net model, recognized for its effectiveness in deep learning applications, particularly in medical image segmentation. The review delved into various U-net variants and their applications across different imaging modalities. Additionally, it assessed major deep learning techniques and their respective fields as discussed in the surveyed literature. The U-net architecture has proven to be innovative and essential for medical image analysis, and the increasing number of publications since 2017 underscores its prominence as a leading deep learning approach in the realm of medical diagnostics [28]. In our review, U-Net variants (Yu et al. and Liefers et al.) [20,25] have been the leading models for fluid segmentation in large cohorts of neovascular AMD with a sample size of 1,097. They achieved strong Dice scores for IRF at 0.70 and SRF at 0.67.20 However, these models required manual corrections for small exudates. A survey aligns with our findings, and a modality-specific survey highlights U-Net’s top ranking for semantic segmentation across 13 AMD biomarkers. It also underscores the ongoing need for the manual refinement of small exudates [29].

Recent reviews of multimodal retinal imaging have highlighted the advantages of combining OCT with Indocyanine Green Angiography (ICGA) to enhance spatial coherence in PCV. Gu et al. discuss the advancements in swept-source OCT angiography (OCTA) for PCV and note that fusion strategies, such as those used in PCV-Net, can improve the delineation of IRF in the Dice Similarity Coefficient (DSC) [30]. However, visualizing sub-RPE structures remains challenging [30]. In our review, PCV-Net (Loo et al.) [21], which is a hybrid of 2D and 3D techniques, effectively combined OCT with indocyanine green angiography (ICGA) to enhance spatial consistency in PCV. This resulted in an increase of +0.43 in the Dice score for IRF segmentation compared to pure 2D models. Nonetheless, its performance for sub-RPE segmentation was still poor, with a Dice score of only 0.11. EfficientNet-b3 and CNNs (Gigon et al.; Szeskin et al.) [24,27] focused on processing speed, achieving results in under 30 seconds per scan for atrophy prediction. However, these models exhibited higher volumetric errors in early lesions, with RORA growth Dice scores ranging from 0.46 to 0.72. Similarly, a study found that CNNs accurately distinguished between cognitive impairment due to Alzheimer's (c-MCI) and stable mild cognitive impairment (s-MCI) in up to 75% of cases, with no significant difference between Alzheimer's Disease Neuroimaging Initiative (ADNI) and non-ADNI images. CNNs show promise for automating diagnosis along the Alzheimer's continuum, requiring no prior feature engineering and performing well across various imaging protocols. This suggests operators can use them without specialized training and are likely applicable to new patient data, potentially facilitating the integration of structural MRI into routine patient assessment and management [31].

Biomarker-Specific Performance and Clinical Utility

Recent advancements in fluid dynamics have demonstrated a strong AI-human agreement, particularly in SRF and IRF, with near-perfect volume correlations. Notably, Mantel et al. [26] reported minimal bias in IRF/SRF volumes, showcasing exceptional reproducibility for IRF. Clinically, residual IRF has been linked to poor visual outcomes, as highlighted by Yu et al. [20], and it was found that monthly anti-vascular endothelial growth factor (anti-VEGF) therapy is more effective than pro re nata (PRN) regimens for fluid reduction, thereby validating hybrid workflows for personalized treatment. In the realm of atrophy and structural biomarkers, Wang et al. [22] achieved nearly perfect quantification of outer segment (OS) volume in retinitis pigmentosa (RP), with a Pearson r of 0.99 and an error of just 0.014 μL through AI with manual correction. However, atrophy metrics presented higher errors, as Szeskin et al. [27] noted a mean difference of +1.25 mm² in the cRORA area, while Gigon et al. [24] found only a moderate correlation (r = 0.52) in RORA growth. This discrepancy reflects findings from Liefers et al. [25], where AI performed well in detecting focal atrophy (with a sensitivity of 86%) but faced challenges in identifying diffuse patterns. Lastly, the assessment of complex pathologies revealed critical gaps, such as with sub-retinal pigment epithelium (RPE) lesions (DSC 0.11; Loo et al.) [21] and volumetric errors of 20.0 nL in pigment epithelial detachments (PED) (Mantel et al.) [26]. Similarly, recent reviews on geographic atrophy (GA) with AI primarily emphasize lesion segmentation, while classification and progression analysis receive comparatively less attention. There is potential for AI to be utilized in additional aspects of GA diagnosis, including examining the significance of hyperfluorescent regions in GA. The application of AI in this context offers numerous benefits, such as enhanced diagnostic precision and quicker processing times [32].

Workflow Efficiency and Clinical Integration

Hybrid OCT workflows can be implemented in both high-volume tertiary centers and community tele-ophthalmology programs. GPU-accelerated OCT pipelines have shown both weak and strong scaling capabilities in local and remote settings, processing tens of thousands of A-scans per second on mid-range GPUs (e.g., RTX 2060) and exceeding 2.6 MHz on high-end graphics cards (e.g., RTX 4090) without requiring any code modifications [33]. In our review, hybrid AI-human workflows consistently demonstrated transformative efficiency gains while enhancing clinical decision-making across all nine studies. The integration of AI for initial segmentation reduced processing time by 50-92% compared to manual grading, with the most dramatic acceleration seen in Loo et al. [23], where MacTel2 cavitation analysis time plummeted from 135.9 to 11.4 seconds per scan, enabling near-real-time quantification during patient visits. Similarly, Wang et al. [22] halved the time required for ellipsoid zone (EZ) and outer segment (OS) segmentation in retinitis pigmentosa, while Mantel et al. [26] achieved full fluid quantification in less than 30 seconds using a fully convolutional neural network with squeeze-excite blocks. These time savings directly translated to enhanced clinical workflows: Hafner et al. [19] leveraged the greater than 50% efficiency gain to implement same-day, biomarker-guided anti-VEGF regimen adjustments in nAMD patients, with IRF volume reductions of 75% (p=0.0198) directly informing dose escalation decisions.

Critically, the integration pathway of AI outputs into clinical care followed three key patterns. The first was automated risk stratification. Gigon et al. [24] transformed AI-generated reticular drusen-associated outer retinal atrophy (RORA) progression risk maps into personalized monitoring schedules, identifying high-risk foveal-ring patterns (Pearson r=0.52 vs. manual), which triggered three-month follow-ups instead of standard six-month intervals. Yu et al. [20] utilized fluid dynamics from 1,097 HARBOR trial eyes to stratify the risk of macular atrophy: every 10μm increase in baseline PED height raised MA odds by 12% (p<0.001), prompting preemptive treatment intensification. The second key pattern involved treatment guidance systems. Liefers et al. [25] embedded automated fluid reports within electronic health records, flagging residual IRF (>0.02μL) associated with significant visual acuity loss. Clinicians used these alerts to modify anti-VEGF intervals in 68% of cases during the study. Similarly, Loo et al. [21] deployed PCV-Net’s ICGA-OCT fusion outputs to guide photodynamic therapy planning, achieving volumetric errors of <0.17μL to ensure accurate targeting of polypoidal lesions. The third pattern was trial endpoint standardization. Wang et al. [22] demonstrated that hybrid EZ area quantification (DSC=0.85 vs. manual) reduced inter-center measurement variability in RP trials by 40%, enabling reliable detection of 0.1-mm² therapeutic effects previously obscured by manual inconsistency. Szeskin et al. [27] achieved vendor-agnostic atrophy monitoring (Topcon/Heidelberg; F1=0.78-0.89), allowing multisite trials to pool data from diverse OCT devices.

The scalability of these workflows was proven in large international studies. Yu et al. [20] processed 1,097 OCT volumes from the HARBOR trial, an effort that would be infeasible with manual grading, revealing that monthly anti-VEGF treatment reduced residual fluid 3.2 times more effectively than PRN regimens (p<0.001). Similarly, Loo et al. [23] analyzed 290 MacTel2 volumes across US and Australian sites, establishing cavitation volume change as a validated trial endpoint with a 24-month volume correlation of r=0.86 vs. manual. Notably, two studies quantified the iterative value of human-AI feedback loops. Mantel et al. (2021) [26] reduced PED segmentation errors by 37% after incorporating clinician corrections into model retraining, while Wang et al. (2023) [22] cut DLM-only EZ area errors by 70% when expanding training data from a smaller to a larger dataset (n=130→400 patients), demonstrating how clinician input scales AI accuracy.

In terms of clinical impact, key workflow innovations were observed across various applications. For nAMD management, implementing same-day fluid-guided treatment resulted in a 75% reduction in IRF and avoided approximately 3.2 delayed treatments per patient, according to Hafner et al. [19]. In RP trial endpoints, cross-device EZ/OS quantification resulted in 40% lower inter-site variability compared to manual methods, alongside a 70% error reduction achieved through scaling, as demonstrated by Wang et al. [22]. For atrophy prevention, AI-predicted risk maps issued by Gigon et al. [24] resulted in 30% shorter monitoring intervals for high-risk foveal-ring progression. In a multicenter fluid analysis, the HARBOR trial-scale processing completed by Yu et al. [20] identified CFRV as a novel biomarker for post-treatment edema (p=0.003). These results validate hybrid workflows as not merely time-saving tools but decision-enabling frameworks that transform quantitative biomarkers into actionable insights -from community clinics to global trials. Future implementation should prioritize seamless EHR integration and resource-optimized workflows, particularly for settings lacking specialization.

Conclusions and Future Directions for AI-Human Collaboration in OCT Pathology Quantification

Hybrid AI-human workflows greatly improve the quantification of OCT pathology, achieving expert-level accuracy for 11 out of 13 biomarkers and reducing processing time by over 50% for conditions such as AMD and RP. While current AI excels at segmenting well-defined features, such as retinal layers and fluid, it still struggles with complex pathologies, such as sub-RPE lesions. Future efforts should focus on developing technical innovations, such as 3D networks and multimodal fusion, to address ambiguous biomarkers; creating adaptive AI systems that learn iteratively from clinicians' corrections; validating these systems in real-world settings across diverse populations; and establishing standardized regulatory frameworks to ensure equitable clinical implementation. Ongoing collaboration between clinicians and AI developers is crucial for transforming quantitative insights into actionable precision medicine. This hybrid paradigm aligns with emerging evidence across medical specialties; recent systematic reviews in oncological imaging demonstrate that AI-human collaboration consistently achieves superior diagnostic performance compared to either AI alone or conventional methods, with accuracy improvements exceeding 20% in complex detection tasks [34].

Strengths and Clinical Implications

Our review comprehensively addresses a significant gap in the ophthalmic AI literature by focusing specifically on hybrid human-AI workflows, marking a crucial shift from fully automated systems. Our methodological strengths include an extensive search strategy across three major databases, employing a tailored syntax for terms related to OCT, AI, and quantification. We implemented a dual-independent review process, encompassing screening, data extraction, and quality appraisal using validated tools such as QUADAS-2 and ROBINS-I, which helped minimize bias in our findings. Importantly, our study emphasizes clinically actionable outcomes, including processing time, volumetric error, and biomarker-specific accuracy, as evidenced by DSC for IRF and SRF. We excluded low-evidence studies, such as conference abstracts and synthetic data, ensuring that only robust evidence supports our conclusions. The clinical implications of our findings are significant: our hybrid workflows can reduce OCT analysis time by more than 50% while maintaining expert-level accuracy for 11 out of 13 biomarkers. This efficiency enables faster treatment decisions for conditions such as neovascular AMD and retinitis pigmentosa (RP). Clinicians can effectively utilize AI for volumetric tracking of fluid and atrophy during anti-VEGF therapy, thereby reserving manual efforts for more complex pathologies, such as sub-retinal pigment epithelial lesions. This collaborative approach not only standardizes quantitative OCT interpretation across clinics and clinical trials but also propels the advancement of precision ophthalmology without sacrificing diagnostic nuance.

Application of AI in Clinical Settings

Hybrid AI-human workflows are revolutionizing clinical OCT analysis by combining the efficiency of AI in quantifying clearly defined biomarkers, such as retinal fluid and layers, with the expertise of clinicians for more complex pathologies, like sub-RPE lesions. This approach has been validated across nine studies and demonstrates a reduction in interpretation time by over 50%. It also minimizes volumetric errors in atrophic regions and achieves expert-level accuracy in 11 out of 13 biomarkers. This directly enhances diagnostic precision for conditions such as neovascular AMD and retinitis pigmentosa. Clinically, these systems facilitate rapid and standardized monitoring of treatment responses in anti-VEGF therapy and enable early detection of disease progression. Moreover, the involvement of clinicians ensures that nuanced judgment can be applied to ambiguous cases. To ensure smooth adoption in real-world settings, future efforts should focus on developing adaptive AI that learns from clinician corrections, creating device-agnostic platforms, and establishing regulatory-compliant workflows that incorporate quantitative outputs into routine diagnostic processes.

Future Recommendations

To enhance the efficacy of AI in biomedical applications, several key strategies should be implemented. First, there is a pressing need to standardize biomarker definitions and training data. Given the observed performance variability, such as in the case of sub-RPE lesions with a DSC of 0.11, establishing consensus definitions for ambiguous pathologies and curating multi-center datasets with unified annotation protocols will significantly improve AI generalizability. Additionally, embedding adaptive learning in hybrid workflows is essential; AI systems should utilize clinician corrections as real-time feedback to iteratively refine segmentation. This approach could leverage the demonstrated time savings of over 50% from reviewed studies to enhance long-term accuracy.

Furthermore, it is crucial to validate AI systems in real-world settings by prioritizing prospective validation in diverse clinical environments, particularly in large-scale trials and resource-limited contexts, to assess robustness across varying populations and OCT devices. Emphasizing complex biomarker innovation is also vital; developers should focus on architectural innovations, such as 3D networks and multimodal fusion, particularly for challenging biomarkers requiring RPE-level precision where current AI performance is lacking. Finally, establishing clear implementation guidelines is necessary for clinicians, AI developers, and regulators to work collaboratively, defining pathways for integrating quantitative AI outputs into diagnostic and therapeutic decision-making, thus ensuring seamless clinical adoption.

Limitations

Heterogeneity among studies, characterized by variable cohort sizes (ranging from 16 to 1,097 patients), diverse OCT devices, and differing definitions of biomarkers like "atrophy," significantly hampers the comparability of findings across research. Additionally, the presence of publication bias likely skews performance metrics, as smaller studies may overrepresent positive outcomes while negative AI results remain underreported. The exclusion of fully automated, non-hybrid AI studies limits access to potentially valuable insights. Furthermore, the generalizability of results is restricted, given that most research has been conducted in academic settings, leaving a gap in validation for real-world primary care. To address these challenges, standardizing biomarker definitions through consensus panels is essential, creating unified OCT pathology criteria akin to medical image computing and computer assisted intervention (MICCAI)-style guidelines. Emphasis should be placed on validating hybrid AI systems in community clinics that utilize a variety of OCT devices and encompass diverse patient demographics. Journals must also promote the reporting of negative results to shed light on AI-human collaboration failures or suboptimal performance. Finally, developing adaptive AI capable of incorporating clinician corrections in real-time could significantly enhance the model's ability to handle complex lesions, such as sub-RPE.

## Conclusions

This systematic review demonstrates that AI-human collaborative workflows for OCT-based retinal pathology quantification achieve high performance for well-defined biomarkers, such as intraretinal fluid and retinal layers, though performance remains variable for complex features, such as sub-RPE lesions. The hybrid approach-where AI handles initial segmentation, and clinicians refine outputs-improves workflow efficiency by over 50% and achieves strong correlation with manual quantification (Pearson r>0.85). With AI exhibiting reliability in 85% of key biomarkers, these systems enable standardized OCT analysis, early detection of neovascular AMD progression, and precise treatment monitoring, successfully combining algorithmic speed with clinical expertise. Current evidence is limited by small cohort sizes and heterogeneous training data. Future priorities include large-scale multi-center validation studies, standardized biomarker definitions, and robust regulatory frameworks to enable safe clinical implementation and establish scalable, data-driven retinal care.
